# Using jackknife to assess the quality of gene order phylogenies

**DOI:** 10.1186/1471-2105-11-168

**Published:** 2010-04-06

**Authors:** Jian Shi, Yiwei Zhang, Haiwei Luo, Jijun Tang

**Affiliations:** 1 Department of Computer Science and Engineering, University of South Carolina Columbia, SC 29028, USA; 2 Department of Biological Sciences, University of South Carolina Columbia, SC 29028, USA

## Abstract

**Background:**

In recent years, gene order data has attracted increasing attention from both biologists and computer scientists as a new type of data for phylogenetic analysis. If gene orders are viewed as one character with a large number of states, traditional bootstrap procedures cannot be applied. Researchers began to use a jackknife resampling method to assess the quality of gene order phylogenies.

**Results:**

In this paper, we design and conduct a set of experiments to validate the performance of this jackknife procedure and provide discussions on how to conduct it properly. Our results show that jackknife is very useful to determine the confidence level of a phylogeny obtained from gene orders and a jackknife rate of 40% should be used. However, although a branch with support value of 85% can be trusted, low support branches require careful investigation before being discarded.

**Conclusions:**

Our experiments show that jackknife is indeed necessary and useful for gene order data, yet some caution should be taken when the results are interpreted.

## Background

Phylogenetic reconstruction is the process to determine the evolutionary histories among organisms. While biologists primarily use DNA or protein sequences to study phylogenies, higher-level rearrangement events such as inversions and transpositions are proving to be useful in elucidating evolutionary relationships. As a result, researchers have used the rearrangement of gene orders to infer high-quality phylogenies [1-4].

Given a set of DNA sequences, we can use procedures such as bootstrap to assign confidence values to edges (branches) in phylogenetic trees [5]. Edges with high confidence values (> 75 - 80%) are generally considered acceptable. However, such procedures are impossible for gene order data since essentially gene orders can be viewed as one character with a very large number of states [6].

Several papers presented a jackknife procedure to overcome the problem [1-3]. However, there are many questions to be answered regarding the performance of jackknife. For example, we need to know how many genes should be removed and how many replicates are needed. We even do not know if jackknife on gene order data will converge. We also need to know above what threshold of confidence values can we claim an edge correct.

In this paper, we conduct a set of experiments to tackle these questions. The remainder of this paper is organized as follows: We first review gene order data and genome rearrangements, along with general bootstrap and jackknife procedures. We then provide details of our experiments. In the Result section, we determine good rates of jackknife, the number of replicates required, and the accuracy of confidence values.

### Gene orders and rearrangements

We assume a reference set of *n *genes {*g*_1_, *g*_2_, ⋯, *g*_*n*_}, and a genome can be represented by an *ordering *of these genes. Each gene is assigned with an orientation that is either positive, written *g*_*i*_, or negative, written -*g*_*i*_. Gene orders can be rearranged through events such as inversions and transpositions. Let *G *be the genome with signed ordering of *g*_1_, *g*_2_, ⋯, *g*_*n*_. An *inversion *between indices *i *and *j *(*i *≤ *j*) produces the genome with linear ordering

The *inversion distance *between two genomes is the minimum number of inversions needed to transform one into the other. Hannenhalli and Pevzner [7] developed a theory for signed permutations and provided a polynomial-time algorithm to compute the edit distance (and the corresponding minimum edit sequence) between two signed permutations under inversions. However, the minimum distance may significantly underestimate the true number of events that have occurred. Several true inversion distance estimators have been proposed and among them, the *EDE *correction [8] is the most used.

There are several widely used methods to reconstruct phylogenies from gene order data, including distance-based methods (neighbor-joining [9] and FastME [10]), Bayesian (Badger [11]) and direct optimization methods (GRAPPA [12] and MGR [13]). Using corrected inversion distances, Wang et al. showed that high-quality phylogenies can be obtained using distance-based methods such as Neighbor-joining and FastME [14]. On the other hand, although Badger, GRAPPA and MGR are more accurate, these methods are computationally very demanding and may not be able to analyze datasets when genomes are distant.

Several other methods have been proposed. For example, MPBE [15] transforms adjacency pairs from the signed permutation into sequence-like strings, while the method proposed by Adam et al. [16] used common intervals (subsets of clusters contiguous in both genomes) to represent gene orders as binary strings. In MPBE, each gene ordering is translated into a binary sequence, where each site from the binary sequence corresponds to a pair of genes. For the pair (*g*_*i*_, *g*_*j*_), the sequence has a 1 at the corresponding site if *g*_*i *_is immediately followed by *g*_*j *_in the gene ordering and a 0 otherwise. These transformed strings are then inputs to the ordinary sequence parsimony software (e.g. PAUP* 4.0 [17]) to obtain a phylogeny. For a complete review, please see [18].

### Bootstrap and jackknife

Bootstrap is commonly used to assess the quality of sequence-based phylogenies. The bootstrap procedure generally starts with creating new alignments by randomly picking alignment columns from the original input alignment and reconstruct a tree independently on each new alignment. A consensus tree is then constructed to summarize the results of all *tree replicates*. The confidence value for an edge in the consensus tree is defined to be the number of replicates in which it appears. If the confidence value for a given edge is 75% or higher, the topology at that branch is generally considered correct.

Although the above bootstrap procedure can be applied to methods such as MPBE where each character of the converted string is treated independently. However, it is not possible to perform this procedure in GRAPPA, MGR and most other methods (except e.g. [15,16]), since for these methods, gene order data can be viewed as one character with 2^*n*^*n*! possible states for genomes with *n *genes [6].

There are several other ways to apply disturbance to gene order data and assess the robustness of the data. For example, one can randomly remove a genome from the dataset or randomly perform a number of events on the gene orders. However, even with 1000 genomes, removal of just one may not introduce enough disturbance. On the other hand, there are many parameters to consider in the latter approach: we need to determine what kind of events to be included, which evolutionary model to use and how to apply the events, how many events to apply, and if we should apply the same amount of events on each genome. Since we still do not have a good evolutionary model for genome rearrangements, it will be difficult to develop an assessment method based on this approach.

Several researchers (including our group) began to use a procedure called jackknife to overcome the problem [1-3].

However, to our knowledge, no detailed study on the performance of this method has been conducted.

In general, the jackknife procedure is performed using the following steps:

• Generating *k *new sets of genomes by deleting some genes. Orders of the remaining genes are preserved with respect to their orders in the original genomes.

• Reconstructing tree replicates from these new genomes.

• Computing a consensus tree and corresponding confidence values on all internal edges.

A consensus tree can be obtained using majority rule, i.e. the consensus only contains edges that exist in more than half of the input trees. The extended majority rule method uses the majority rule result as a start and greedily adds edges that occur in less than half of the input trees, with the aim that a full binary tree can be obtained. In this paper, we use the CONSENSE program in PHYLIP [19]. We find that the extended majority rule consensus trees generally outperform those computed with majority rule.

## Results

### Determining jackknife rate

Indeed, jackknife has been used for sequence data before, although it is not as common as bootstrap. Felsenstein suggested for DNA sequences, that "one way to make the jackknife vary as much as the boot-strap would be to delete half of the characters, at random, in each replicate [5]." Farris later stated that 50% deletion is too severe [20] and suggested the rate of 1/*e *≈ 37% should be used. The jackknife rate (how many genes should be deleted) is critical for gene order data as well: leaving too few genes out would not produce enough disturbances to the original data, while removing too many genes would make the data totally unrecognizable. The jackknife rate of 50% was adopted by the limited number of papers where jackknife were used [1-3]. However, no discussion was given on the choice of such rate.

**Figure 1:**
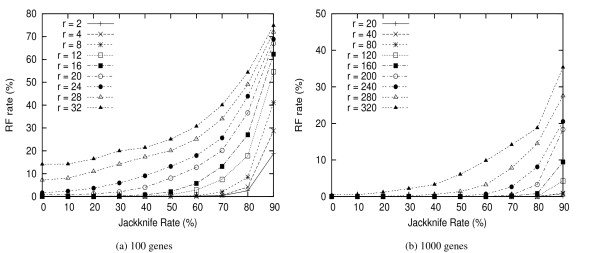
**Jackknife Rate**. The RF rates on different jackknife rates (*r *is the expected number of events per edge). A jackknife rate of 10% means only 10% genes are removed.

To determine the good jackknife rates, we conduct the following experiments: Given a dataset, we choose the jackknife rate from 0% (no gene is deleted) to 90% (9 out of 10 genes are deleted) and run 100 replicates on each rate. We then use FastME to reconstruct a phylogeny tree for each replicate. For each rate, we obtain a consensus tree and compare it with the true tree. The above procedure is repeated for all datasets, and the average RF rates [21] are shown in Figure 1.

**Figure 2:**
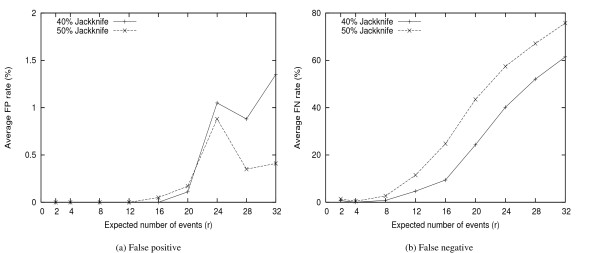
**Comparison between 40% and 50% Jackknife Rates**. FP and FN rates of the inferred trees using 40% and 50% jackknife rates, by contracting edges with < 85% confidence value.

We find from Figure 1 that the jackknife rates of 40% and 50% produce similar results. To determine which one is better, we make further investigation on the quality of inferred trees by removing low supporting branches (< 85% confidence value) from the consensus trees. Figure 2 shows the results from datasets with 100 genes; the measurements are false negatives (FP) and false positives (FN) errors [21]. In this figure, both 40% and 50% rates produce trees with very low FP errors (< 2%) and the results are comparable: 40% has slightly better performance for lower evolutionary rates (*r *< 24), while 50% is better for *r *≥ 24. However, using 50% jackknife rate generates much higher FN errors for all datasets, especially when *r *< 24. Based on this comparison, we use the rate of 40% in all our other experiments.

### Number of replicates required

In [1-3], the authors used 100 replicates to obtain the confidence values, following traditions in bootstrap. Pattengale et al. [22] discussed the number of replicates for DNA bootstrap and conducted a complete research about finding the correct number of bootstrap replicates. They found that this number indeed varies in a big range. To find out the requirement of replicates in gene order data, we conduct similar testing:

• For a given dataset, generate *k *replicates using jackknife rate of 40%, starting from *k *= 50.

• Randomly split the *k *replicates into two equal sized subsets *s*_1 _and *s*_2_, each containing *k/*2 replicates.

• Compute a consensus tree *t*_1 _from subset *s*_1 _and compare it with the consensus tree *t*_2 _obtained from *s*_2_.

• Stop if *t*_1 _and *t*_2 _are very close; otherwise, increase *k *by 50 and repeat the above procedures.

We use the Weighted Robinson-Foulds (WRF) [23] distance to determine the difference between *t*_1 _and *t*_2_. The WRF distance can be computed as following: For two consensus trees *t*_1 _and *t*_2_, assume *t*_1 _has *N*_1 _bipartitions and *t*_2 _has *N*_2 _bipartitions, and the confidence value for each bipartition is 0 ≤ *w *≤ 100%. Let *W*_1 _be the summation of the confidence values of all the bipartitions in *t*_1 _that are not in *t*_2 _and *W*_2 _be the summation of the confidence values of all the bipartitions in *t*_2 _that are not in *t*_1_. The WRF distance is then

**Figure 3:**
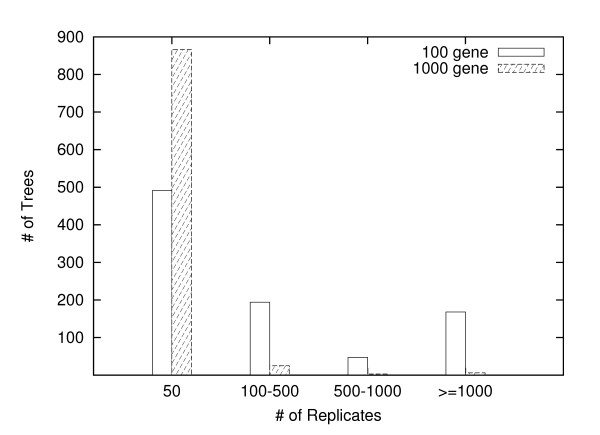
**Converging point distribution**. The distribution of number of replicates required to converge.

To minimize the variation of results due to random splitting, we repeat the above process for 100 times, and calculate the average WRF distance between *t*_1 _and *t*_2_. If this distance is small enough (we use a threshold of 0.03 for consistency with the methodology of [22]), we can assume that enough amounts of jackknife replicates are generated because we keep getting the same consensus trees from different splits. Otherwise, we have to increase *k *and repeat the process until we achieve a satisfying average WRF distance. We call the jackknife procedure converging when there is no need to add more replicates, and the final value of *k *is called the converging point for that dataset. Figure 3 shows the distribution of converging points. For the 900 datasets with 100 genes, about 50% trees require only 50 replicates to converge, while about 30% datasets require more than 500 replicates. For datasets with 1000 genes, almost all datasets require only 50 replicates. These experiments suggest that similar to sequence data, using jackknife on gene order data should choose a different number of replicates for each dataset, and 100 replicates may not be enough for many datasets, especially when the genomes are small. We also notice some datasets require a very large number of replicates to converge (> 3000). These datasets all have very large pairwise distances (close to saturate), thus FastME is not very accurate, making the jackknife procedure hard to converge.

### Threshold of confidence values

The confidence values of internal edges are perhaps the most valuable information obtained through the jackknife procedure. However, as in bootstrap, the meaning of these values is always up for interpretation. The most important question is to determine where to draw the threshold so that edges with confidence values higher than this threshold can be trusted, whereas edges with lower values can be discarded.

We design the following experiments to find out a good threshold value:

• For each dataset, determine its converging point *k *and compute a consensus tree on these *k *replicates.

• For a given threshold value *M*, contract all edges with confidence values below *M*.

• Compare the true trees with the contracted trees to obtain FP and FN rates.

• Repeat the above procedures for 60 ≤ *M *≤ 95.

**Figure 4:**
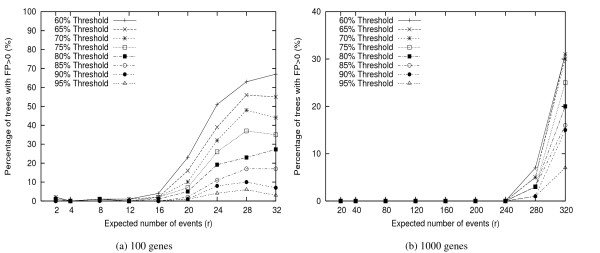
**False Positive Tree Rate**. The percentage of trees with FP branches > 0, under different threshold values.

Figure 4 shows the percentage of trees that have false positive edges. We are more interested in FP branches because they were not in the true tree and should be identified by the jackknife procedure. Not surprisingly, from this figure we find that fewer than 20% trees have FP for large threshold values (*M *≥ 85) even under very high *r *value.

However, the FN rates are very high for these low thresholds, especially when the genomes are distant.

**Figure 5:**
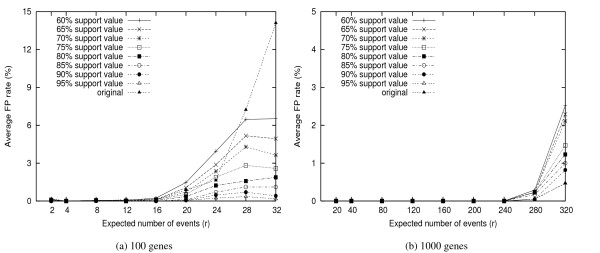
**Average FP Rate**. The FP branch rates under different threshold values. The results of the original datasets are the comparison of the true tree with the phylogeny obtained from the original genomes.

**Figure 6:**
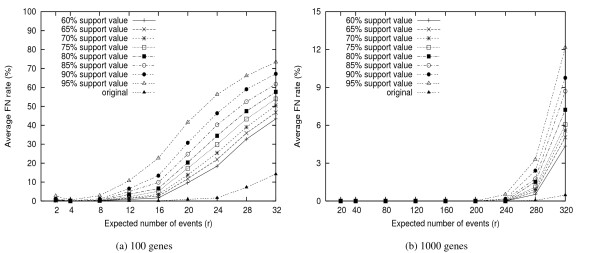
**Average FN Rate**. The FN rates under different threshold values. Very large FN rates are observed when the threshold values are too high.

Figures 5 and 6 show the average FP and FN rates respectively for different threshold values, with comparison to the FP(FN) rates of the phylogenies obtained from the original genomes, i.e. the genomes without removing any gene. We observe that by doing jackknife, about 95% bad edges can be identified if the threshold value is set at 85%. In other words, jackknife is very much needed for gene order phylogeny study.

By comparing all values presented in Figures 4 to 6, we suggest the use of threshold value of 85%, which results in the best balance of FP and FN. Under the extreme case, using *M *= 85%, almost 50% *true *branches can be resolved with only 10% chance of error, and the expected FP rates are ≤ 3%.

**Figure 7:**
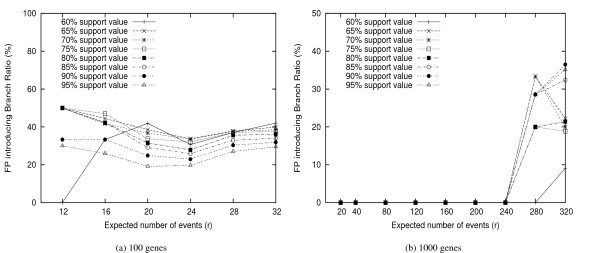
**False Positive introducing Branch Rate**. The percentage of low support branches that actually are false positive. No FP is reported given *r *≤ 12 for 100 gene datasets. For 1000 gene datasets, no FP when *r *≤ 240.

However, the high FN rates may reflect that too many potentially good edges are discarded due to low confidence values. To identify how many of such branches are wasted, we check each low support edge and determine if it is indeed a false positive. Figure 7 shows the percentage of such mistakenly discarded edges, under different threshold values. We are surprised to find that for *M *= 85%, almost two thirds of branches are not used due to low confidence values, although these branches occur in the true tree, and thus should not be thrown out. These errors may be introduced by the phylogenetic methods (FastME), the consensus method, or the jackknife procedure itself. (In Figures 5 and 6, we can see that FP and FN rate are around 15% even for the original data without being jackknifed.) Further investigations are needed to reduce these errors to improve the performance of jackknife.

## Methods

In this paper, we concentrate our experiments on simulated datasets so that the quality of jackknife replicates can be assessed against the known true tree. In our simulations, we generate model tree topologies from the uniform distribution on binary trees, each with 20 leaves. On each tree, we evolve signed permutations of 100 and 1000 genes using various numbers of evolutionary rates: letting *r *denote the expected number of inversions along an edge of the true tree, we use values of *r *= 2, 4, 8, ⋯, 32 for 100 genes and *r *= 20, 40, 80, ⋯, 320 for 1000 genes. The actual number of inversions along each edge is sampled from a uniform distribution on the set . For each combination of parameter settings, we run 100 datasets and average the results.

We always use FastME to obtain phylogenies since it is very accurate with corrected inversion distances [14]. Other methods (GRAPPA and MGR) will take very long time for datasets with 20 genomes and large *r *values.

We assess topological accuracy via *false negatives *and *false positives *[21]. Let *T *be the true tree and let *T' *be the inferred tree. An edge *e *in *T *is "missing" in *T' *if *T' *does not contain an edge defining the same bipartition; such an edge is called a *false negative *(FN). The *false negative rate *is the number of false negative edges in *T' *with respect to *T *divided by the number of internal edges in *T*. The *false positive (FP) rate *is defined similarly, by swapping *T *and *T'*. The *Robinson-Foulds *(RF) rate is defined as the average of the FN and FP rates. An RF rate of more than 5% is generally considered too high [24].

## Conclusions

We have conducted extensive experiments to validate the performance of jackknife on gene order phylogenies. These testings show that jackknife is very useful to determine the confidence level of a phylogeny, and a jackknife rate of 40% should be used. However, although a branch with support value of 85% can be trusted, low support branches should not be discarded without further investigation. The jackknife rate of 40% is very close to the suggested rate of 37% for sequence data [20], thus we need to conduct theoretical analysis on the foundation of jackknife on genome rearrangements. All our experiments are conducted with FastME, experiments using other methods should be conducted to further evaluate the performance of jackknife.

## Competing interests

The authors declare that they have no competing interests.

## Authors' contributions

All authors contributed to the development and implementation of the methods. JS, YZ and JT were in charge of conducting simulations and analyzing results. All authors read and approved the final manuscript.

## References

[B1] Belda E Moya A Silva F Genome rearrangement distances and gene order phylogeny in γ-Proteobacteria Mol Biol Evol 2005 22 1456 1467 10.1093/molbev/msi134 15772379

[B2] Luo H Shi J Arndt W Tang J Friedman R Gene order phylogeny of the genus Prochlorococcus PLoS ONE 2008 3 e3837 10.1371/journal.pone.0003837 19050756 PMC2585141

[B3] Luo H Sun Z Arndt W Shi J Friedman R Tang J Gene order phylogeny and the evolution of Methanogens PLoS ONE 2009 4 e6069 10.1371/journal.pone.0006069 19562076 PMC2699033

[B4] Raubeson L Jansen R Chloroplast DNA evidence on the ancient evolutionary split in vascular land plants Science 1992 255 1697 1699 10.1126/science.255.5052.1697 17749424

[B5] Felsenstein J Confidence limits on phylogenies: An approach using the bootstrap Evolution 1985 39 783 791 10.2307/2408678 28561359

[B6] Moret B Warnow T Advances in phylogeny reconstruction from gene order and content data Methods in Enzymology 2005 395 673 700 full_text 15865990 10.1016/S0076-6879(05)95035-0

[B7] Hannenhalli S Pevzner P Transforming cabbage into turnip (polynomial algorithm for sorting signed permutations by reversals Proceedings of the 27th Ann Symp Theory of Computing (STOC'95) 1995 99 124

[B8] Moret B Wang L Warnow T Wyman S New approaches for reconstructing phylogenies based on gene order Proceedings of the 9th Intl Conf on Intel Sys for Mol Bio (ISMB'01) 2001 165 173 10.1093/bioinformatics/17.suppl_1.s165 11473006

[B9] Saitou N Nei M The neighbor-joining method: A new method for reconstructing phylogenetic trees Mol Biol Evol 1987 4 406 425 3447015 10.1093/oxfordjournals.molbev.a040454

[B10] Desper R Gascuel O Fast and accurate phylogeny reconstruction algorithms based on the minimum evolution principle J Comput Biol 2002 9 687 705 10.1089/106652702761034136 12487758

[B11] Larget B Kadane J Simon D A Bayesian approach to the estimation of ancestral genome arrangements Mol Phy Evol 2005 36 214 223 10.1016/j.ympev.2005.03.026 15893477

[B12] Moret B Wyman S Bader D Warnow T Yan M A new implementation and detailed study of breakpoint analysis Proceedings of the 6th Pacific Symp on Biocomputing (PSB'01) 2001 583 594 10.1142/9789814447362_0056 11262975

[B13] Bourque G Pevzner P Genome-scale evolution: reconstructing gene orders in the ancestral species Genome Research 2002 12 26 36 11779828 PMC155248

[B14] Wang L Jansen R Moret B Raubeson L Warnow T Distance-based genome rearrangement phylogeny J Mol Evol 2006 63 473 483 10.1007/s00239-005-0216-y 17021931

[B15] Wang L Jansen R Moret B Raubeson L Warnow T Fast phylogenetic methods for genome rearrangement evolution: An empirical study Proceedings of the 7th Pacific Symp on Biocomputing (PSB'02) 2002 Hawaii: World Scientific Pub 524 535 11928504

[B16] Adam Z Turmel M Lemieux C Sankoff D Common intervals and symmetric difference in a model-free phylogenomics, with an application to streptophyte evolution J Comput Biol 2007 14 436 445 10.1089/cmb.2007.A005 17572022

[B17] Swofford D PAUP*. Phylogenetic analysis using parsimony (*and other methods). Version 4 Sunderland, MA 2003

[B18] Fertin G Labarre A Rusu I Tannier E Vialette S Combinatorics of genome rearrangements 2009 The MIT Press

[B19] Felsenstein J PHYLIP-Phylogeny Inference Package Cladistics 1989 5 164 166

[B20] Farris J Albert V Kallersjo M Lipscomb D Kluge A Parsimony jackknifing outperforms neighbor-joining Cladistics 1996 12 99 124 10.1111/j.1096-0031.1996.tb00196.x 34920604

[B21] Robinson D Foulds L Comparison of phylogenetic trees Mathematical Biosciences 1981 53 131 147 10.1016/0025-5564(81)90043-2

[B22] Pattengale N Alipour M Bininda-Edmonds O Moret B Stamatakis A How many bootstrap replicates are necessary? Proceedings of the 13th Int'l Conf on Research in Comput Molecular Biol (RECOMB'09) 2009 184 200 10.1089/cmb.2009.0179 20377449

[B23] Robinson D Foulds L Comparison of weighted labeled trees Combinatorial Mathematics VI 1979 748 119 126 full_text

[B24] Swofford D Olson G Waddell P Hillis D Hillis D, Moritz C, Mable B Phylogenetic inferences Molecular Systematics 1996 2

